# Comparison among nasopharyngeal swab, nasal wash, and oropharyngeal swab for respiratory virus detection in adults with acute pharyngitis

**DOI:** 10.1186/1471-2334-13-281

**Published:** 2013-06-20

**Authors:** Li Li, Qiao-Yan Chen, Yun-Ying Li, Yan-Fang Wang, Zi-Feng Yang, Nan-Shan Zhong

**Affiliations:** 1 Guangzhou Institute of Respiratory Disease, State Key Laboratory of Respiratory Diseases (Guangzhou Medical University), The First Affiliated Hospital of Guangzhou Medical University, 151 Yanjiang Road, Guangzhou 510120, China; 2 Guangdong Provincial Hospital of Chinese Medicine, The Second Clinical College of Guangzhou University of C.M., Guangdong Provincial Academy of Chinese Medical Sciences, 111 Da De Road, Guangzhou 510120, China; 3 South China Normal University, 55 Zhong Shan Road West, Guangzhou 510631, China

**Keywords:** Respiratory viruses, Acute pharyngitis, Oropharyngeal swab, Nasopharyngeal swab, Nasal wash, Sensitivity, TaqMan real-time polymerase chain reaction

## Abstract

**Background:**

Acute pharyngitis is frequently seen in primary care. Acute viral pharyngitis may be easily misdiagnosed as acute bacterial pharyngitis. Laboratory-confirmed diagnosis of respiratory viruses is recommended. The purpose of this study was to compare the sensitivities among oropharyngeal swab (OPS), nasopharyngeal swab (NPS), and nasal wash (NW) in adults with acute pharyngitis.

**Methods:**

OPS, NPS, and NW were obtained from each participant with acute pharyngitis. The specimens were tested for 15 respiratory viruses by TaqMan real-time polymerase chain reaction. A sample was considered to be a true positive if any of the specimens was positive. The sensitivities among samples were compared by chi-square test or Fisher’s exact test, as appropriate.

**Results:**

One hundred three triple samples collected consecutively by OPS, NPS, and NW were obtained. In 73 patients, one or more viruses were detected by any of the three methods. Among all viruses, the sensitivity of NPS was significantly higher than that of NW (74% vs. 49%, respectively; p < 0.01) and OPS (74% vs. 49%, respectively; p < 0.01).

**Conclusions:**

Flocked NPS collection may be the most effective alternative to NW and OPS for detection of respiratory viruses in adults with acute pharyngitis using TaqMan real-time polymerase chain reaction.

## Background

Acute pharyngitis is one of the most common illnesses for which patients visit primary care physicians [1], accounting for 1% to 2% of all patient visits [2]. In adults, its etiology is primarily viral; only about 10% of patients have a bacterial cause, most commonly a beta-hemolytic streptococcus [1]. Despite the high predominance of viruses over any other cause in adults [3,4], physicians prescribe antibiotics for 78% to 98% of patients [5,6]. A laboratory-confirmed diagnosis of respiratory viruses could support the appropriate use of antiviral therapy and reduce the inappropriate use of antibiotics for patients. However, bacteriological investigation in general practice is not always performed [7], and virological investigation is rarely done. Acute pharyngitis is characterized by inflammation of the oropharyngeal cavity and surrounding lymphoid tissue [8]. Inflammation manifests as pain of varying intensity. An acutely sore throat is the premonitory symptom of many acute infectious diseases, including acute infectious mononucleosis [9]. Moreover, acute pharyngitis is associated with complications such as otitis media, sinusitis, and upper and lower respiratory tract infections [10,11]. Thus, virological investigation prior to treatment should be considered for patients with acute pharyngitis. This approach would also be the most cost-effective for patients.

The identification of respiratory viruses in patient samples is highly dependent on the source of the clinical specimens [12]. The nasal wash (NW) has generally been considered to be superior to swab specimens for detection of respiratory viruses [13,14]. However, obtaining a wash may be unpleasant to the patient and requires specialized training and equipment for the provider. In contrast, swab samples are easier to collect and may be preferred by healthcare providers. To date, few studies have compared sampling techniques for virus detection in adults with acute pharyngitis. This study aimed to compare the sensitivities among oropharyngeal swab (OPS), nasopharyngeal swab (NPS), and NW in adults with acute pharyngitis using TaqMan real-time polymerase chain reaction (PCR).

## Methods

### Participants

Between December 2011 and December 2012, we recruited 103 patients from the First Affiliated Hospital of Guangzhou Medical University who had presented with complaints of a sore throat and/or odynophagia within the preceding 3 days. We used the McIsaac scoring system [15] to exclude bacterial throat infections. Complete inclusion and exclusion criteria are listed in Table 1.

**Table 1:** Inclusion and exclusion criteria

**Inclusion and exclusion criteria**	
**Inclusion**	
•	18 years ≤ Age < 60 years
•	History of sore throat and/or odynophagia ≤ 3 days
•	≤ 1 McIsaac score
**Exclusion**	
•	History of sore throat and/or odynophagia > 3 days
•	Symptoms of sore throat and/or odynophagia caused by local irritation of mucous membranes as a result of gastroesophageal reflux

### Collection and storage of material

Experienced physicians collected three consecutive samples from each patient with acute pharyngitis in the following order: OPS, NPS, and NW. The physicians were specifically trained for the task. For OPS, a swab with a rayon tip and plastic applicator (167KS01; Copan Italia S.p.A., Brescia, Italy) was used to obtain an oropharyngeal sample of the posterior oropharyngeal mucosal membrane. For NPS, a flexible flocked swab with a nylon tip and plastic applicator (503CS01; Copan Italia S.p.A.) was inserted into one of the nostrils until slight resistance was felt at the nasopharynx. The swab was then rotated two to three times, held in place for 5 seconds, and withdrawn. The NW was performed after 5 ml of sterile normal saline had been injected into the other nostril by a 5-ml syringe with a nasal adapter (patients were asked to avoid swallowing), then immediately sucked out. The NW process was repeated in the other nostril if less than 2 ml of fluid was retrieved [16]. After sampling, the OPS and NPS applicators were cut off and inserted separately into two tubes containing 1 ml of viral transport medium (Copan Diagnostics Inc., Brescia, Italy). The washes were placed into a sterile container for transport to the laboratory. The specimens were stored at 4°C within 6 hours until shipment to the State Key Laboratory of Respiratory Disease, where they were stored at −80°C until testing.

### Testing for respiratory viruses

The NP and OPS samples were first vortexed, then centrifuged. All supernatant and cellular materials from the samples were used for further testing. The NW handling method was similar to that of NP and OPS, but only 1 ml of supernatant was retained to resuspend the cellular material, and the remainder was discarded. A total of 200 μl of each sample (OPS, NPS, and NW) was extracted using QIAamp MinElute Virus Spin kit (Qiagen China [Shanghai] Co., Ltd.) according to the manufacturer’s instructions. Fifteen common respiratory tract pathogens were tested by TaqMan real-time PCR as previously reported [17], including influenza virus (A and B), parainfluenza virus (PIV 1, 2, 3, and 4), rhinovirus, respiratory syncytial virus (RSV), adenovirus, enterovirus (EV), human coronavirus (HCoV-229E, OC43, NL63, and HKU1), and human metapneumovirus (HMPV). Specimens with a cycle threshold of ≤35 were regarded as positive, and specimens with a cycle threshold of >35 were regarded as negative in accordance with the manufacturer’s protocol (Guangzhou HuYanSuo Medical Technology Co., Ltd.).

### Ethics

This study was conducted with approval of the First Affiliated Hospital of Guangzhou Medical University Ethics Committee, and informed consent was obtained from all patients.

### Statistical methods

We assessed the sensitivity of each sampling method by considering any positive result from each of the specimens as a true positive. We compared the sensitivities using the chi-square test or Fisher’s exact test, as appropriate. Statistical significance was concluded if the p-value was <0.05. Data were recorded and analyzed with the software SPSS version 17.0.

## Results

During the study period, 103 patients with acute pharyngitis were enrolled. The mean age was 28.9 years (SD, 8.3 years), and 42% of patients were men. The mean number of days from onset of illness to sample collection was 2.2 (SD, 0.8). At least one type of virus was detected in 73 patients (73/103, 71%). NPS, NW, and OPS identified infections in 59 (59/103, 57%), 38 (38/103, 37%), and 44 (44/103, 43%) patients, respectively.

For detection of all viruses, the sensitivity of NPS was significantly higher than that of NW (74% [95% CI = 65–83] vs. 49% [95% CI = 39–60], respectively; p < 0.01) and OPS (74% [95% CI = 65–83] vs. 49% [95% CI = 39–60], respectively; p < 0.01). For detection of rhinovirus, the sensitivity of NPS was significantly higher than that of NW (100% [95% CI = 78–100] vs. 60% [95% CI = 32–84], respectively; p < 0.05) and OPS (100% [95% CI = 78–100] vs. 67% [95% CI = 38–88], respectively; p < 0.05). NPS also was significantly more sensitive than NW for detecting adenovirus (75% [95% CI = 43–95] vs. 17% [95% CI = 2–48], respectively; p < 0.05). For coronavirus, influenza virus, EV, RSV, PIV, and HMPV, the differences among NPS, NW, and OPS were not statistically significant (Table 2).

**Table 2:** Comparison of frequency distribution and sensitivity for total viruses detected by triple method

	**Total**	**NPS**		**NW**		**OPS**	
		**No. identified**	**Sensitivity (95%CI**^**a**^**)**	**No. identified**	**Sensitivity (95%CI)**	**No. identified**	**Sensitivity (95%CI)**
**Coronavirus**	29	19	66 (46-82)	17	59 (39-76)	17	59 (39-76)
**Influenza**	19	13	68 (43-87)	10	53 (29-76)	9	47 (24-71)
**Rhinovirus**	15	15	100 (78-100) ^b^	9	60 (32-84)	10	67 (38-88)
**Adenovirus**	12	9	75 (43-95)^c^	2	17 ( 2-48)	4	33 (10-65)
**EV**	7	5	71 (29-96)	2	29 ( 4-71)	1	14 ( 0-58)
**RSV**	3	1	33 ( 1-91)	2	67 ( 9-99)	1	33 ( 1-91)
**PIV**	3	3	100 (29-100)	1	33 ( 1-91)	1	33 ( 1-91)
**HMPV**	1	1	100 ( 2-100)	1	100 ( 2-100)	1	100 ( 2-100)
**All viruses**	89	66	74 (65-83)^d^	44	49 (39-60)	44	49 (39-60)

^a^*CI* confidence interval.

^b^ p < 0.05 versus the results for NW and versus OPS.

^c^ p < 0.05 versus the results for NW.

^d^ p < 0.01 versus the results for NW and versus OPS.

## Discussion

Acute pharyngitis is frequently seen in primary care [1]. Acute viral pharyngitis may be easily misdiagnosed as acute bacterial pharyngitis. Laboratory-confirmed diagnosis of respiratory viruses is recommended. However, few studies focusing on respiratory virus detection in adults have been conducted [18]. Data on the comparison of different sampling methods for respiratory virus detection in adults with acute viral pharyngitis are rare.

This study compared the sensitivities among NPS, OPS, and NW. To exclude patients with bacterial infection and increase the viral detection rate, only patients with a McIsaac score of ≤1 participated in the study. Because NPS followed by NW in the same nostril may reduce the number of cells collected by NW and reduce the sensitivity of the assay, NPS and NW were performed in different nostrils [19]. TaqMan real-time PCR was used to detect common respiratory viruses. In the past, viral culture was considered the “gold standard” method for viral detection, but the turnaround time of traditional culture is generally too long to be clinically feasible [20]. PCR offers both a substantially higher test sensitivity and a more rapid turnaround time [21,22].

A variety of sample collection techniques are used to detect respiratory viruses, including NPS, OPS, nasal aspiration, NW, nasal swab, and sputa and saliva evaluation. NW and aspiration have generally been considered to be superior to swab specimen evaluation for the detection of respiratory viruses [13,23-25]. On the contrary, a study by Patrick et al. found that NPS had a higher sensitivity than NW for detection of viruses by real-time PCR in children [26]. In addition, a study by Agoritsas et al. showed that NPS and nasal swab were superior to nasopharyngeal wash for rapid immunoassay, and that both can be recommended as alternative collection methods to nasopharyngeal wash [27].

In previous studies, many authors have used different collection methods to identify EV (throat swab) [28], HMPV (nasal swab) [29], rhinovirus (nasal and throat swab) [30], influenza (throat and nasal swab) [31], and RSV (nasopharyngeal aspirate and nasal swab) [19]. Moreover, Moës et al. used bronchoalveolar lavage, pharyngeal swabs, nasopharyngeal aspirates, and sputum samples for the identification of coronavirus, although the study did not aim to compare the efficacy of sampling methods [32]. In some clinical studies, two or more virus types were detected by different sampling methods; for example, throat swabs [17], NW [33], nasopharyngeal aspirates [34], or nasal swabs [35]. So far, the differences in the efficacy of various sampling methods are unclear. The paucity of this type of study among the adult population indicates that the same sampling methods have lower sensitivities for adults than for children and adolescents [36,37]. Furthermore, different sampling methods can affect the results of laboratory testing.

Our findings demonstrated that NPS yielded the highest sensitivity among the three sampling methods. For rhinovirus, NPS had a statistically higher sensitivity than NW and OPS. For adenovirus, NPS had a statistically higher sensitivity than NW. In contrast, NW and OPS produced lower sensitivities of viral detection. The prevalence of influenza virus, EV, RSV, PIV, and HMPV was lower than that of rhinovirus. Although our study was not able to compare the differences among these viruses, the order of the sensitivities tended to be the same in the majority of and in the total viruses. A larger sample size may be needed to determine the significance of these differences. In addition, the study was conducted during a whole year comprising different seasons, which experienced the low influenza disease activity in Guangzhou. The seasonality of coronavirus and adenovirus was similar to that in the previous year in China [38-40]. Furthermore, our results are consistent with the finding of Munywoki et al., who showed that nasopharyngeal flocked swab was significantly more sensitive than NW collection for detection of viruses by real-time multiplexed PCR in pediatric patients [26]. Therefore, the data in this study are informative and provide a reference for appropriate collection methods, particularly in subtropical cities.

The choice of a specimen should be based on the efficiency of viral detection, cost, and available expertise. For all viruses detected in the present study, NPS yielded the highest sensitivity. Moreover, compared with NW, NPS is less invasive and more acceptable to patients. NPS can be easily implemented in physicians’ clinics or emergency rooms because the collection process is rapid, little training of personnel is needed, and no special instrumentation is required [12].

This study has a limitation. The NPS material was different from the OPS material. Studies have demonstrated that the flocked swab design yields significantly more total respiratory epithelial cells and more infected respiratory epithelial cells than does the conventional rayon swab; it also provides adequate numbers of respiratory epithelial cells for diagnosis, whether using oropharyngeal samples or nasopharyngeal samples [41-44]. Nasopharyngeal samples reportedly have advantages over oropharyngeal samples for the identification of common respiratory viruses [25,45]. In China, the collection of oropharyngeal samples is mainly performed using the conventional rayon swab. Because of the relatively higher cost of the rayon swab, the flocked swab is seldom used to obtain nasopharyngeal samples. Therefore, oropharyngeal samples were obtained using rayon swabs and nasopharyngeal samples were obtained using flocked swabs in our study.

## Conclusions

Some studies have recommended collection with a combination of two or more sampling methods as the most effective approach [45,46]However, the use of multiple sampling methods increases cost and wastes medical resources. Therefore, we conclude that flocked NPS collection may be the most effective alternative to NW and OPS for detection of respiratory viruses in adults with acute pharyngitis using TaqMan real-time PCR.

## Abbreviations

CI: Confidence interval; EV: Enterovirus; HMPV: Human metapneumovirus; NPS: Nasopharyngeal swab; NW: Nasal wash; OPS: Oropharyngeal swab; PCR: Polymerase chain reaction; PIV: Parainfluenza virus; RSV: Respiratory syncytial virus.

## Competing interests

The authors declare that they have no competing interests.

## Authors’ contributions

Z-FY and N-SZ designed the study. LL and Q-YC collected the clinical data. Y-YL and Y-FW performed the laboratory work. All authors participated in the data analysis. LL and Q-Y C drafted the manuscript. All authors read and approved the final manuscript.

## Authors’ information

Li Li and Qiao-Yan Chen are co-first authors.

## Pre-publication history

The pre-publication history for this paper can be accessed here:

http://www.biomedcentral.com/1471-2334/13/281/prepub
